# Substance Use Disorder and Cognitive Impairment

**DOI:** 10.3390/life16091443

**Published:** 2026-08-30

**Authors:** Wei Li, Isaiah Chamoun, Suruchi Ghaiye, Vy Ha, Rebecca Billings

**Affiliations:** 1 Department of Clinical and Diagnostic Sciences, School of Health Professions, University of Alabama at Birmingham, Birmingham, AL 35294, USA; 2 Department of Genetics, Heersink School of Medicine, University of Alabama at Birmingham, Birmingham, AL 35294, USA; 3 Department of Clinical, Academic, & Research Engagement, Lister Hill Library of Health Sciences, University of Alabama at Birmingham, Birmingham, AL 35294, USA

**Keywords:** cognition, cognitive impairment, substance use disorder (SUD)

## Abstract

(1) Substance use disorder (SUD) is a global issue, which affects people for their physical and mental health. However, it remains unclear about how SUD is associated with cognitive impairment. (2) A literature search was performed across PubMed, Web of Science, and Google Scholar using keywords: cognitive impairment, cognitive dysfunction/prevention and control, behavior, addictive/prevention and control, substance use disorder, addictive/rehabilitation, and recovery. Data from 20 original research studies were extracted to analyze how SUD is associated with cognitive functions. (3) All substances, including tobacco, are associated with a measured decrease in cognitive function in at least one domain. The longer the duration of substance use, especially if multiple substances were used concurrently, the worse the cognitive performance. Abstinence, targeted medication, or cognitive remediation therapy can be used to treat SUD and associated cognitive impairments. (4) Cognitive function should be a focus for rehabilitation programs. For people with SUD, cognitive impairments may hinder them from seeking available treatment options. On the other hand, improved cognitive function may increase the possibility of successful rehabilitation for people with SUD.

## 1. Introduction

Substance use disorder (SUD), which often presents with a set of addiction behaviors, affects people globally on their physical and mental health. For people with SUD, they are unable to control the use of legal or illegal substances like alcohol, tobacco, and drugs despite harmful consequences. In 2023, it was estimated there were 316 million illicit drug users (excluding alcohol and tobacco users) around the world, underscoring the global prevalence of SUD [1]. The symptoms and effects of SUD vary based on the amount and type of the used substance as well as duration, dose (frequency of use), age, and mode of administration. The two typical groups of symptoms for people with SUD include intoxication and withdrawal effects of the substance. Compared with mental health issues, the cognitive function changes associated with SUD are less commonly assessed [2].

Cognitive impairment refers to a condition in which the brain function declines in one or more domains including memory, language, processing speed, etc. The degree of cognitive impairment varies from mild to the most severe form: dementia. Neuropsychological assessments are often used to evaluate cognition and determine if a person has cognitive impairment or not. The most used cognitive assessment tools in clinical settings are the Mini-Mental State Examination (MMSE) and Montreal Cognitive Assessment (MoCA) [3]. MoCA evaluates seven cognitive domains, including visuospatial and executive function, naming, memory, attention, language, abstraction, and orientation [3]. For the MoCA, a cutoff score of below 23 is the most accurate for ascertaining cognitive impairment [4]. By contrast, the cutoff score for MMSE to diagnose mild cognitive impairment (MCI) is higher than 23 although it varies from study to study. Further, the MoCA was found to be more sensitive than the MMSE for detecting MCI [5].

The current study investigates the relationship between SUD and cognitive impairment. Our primary goal is to better understand how SUD impacts cognition. In addition, our study also seeks evidence on the effects of prevention strategies or targeted treatments on SUD especially on the cognition.

## 2. Methods

**Figure 1:**
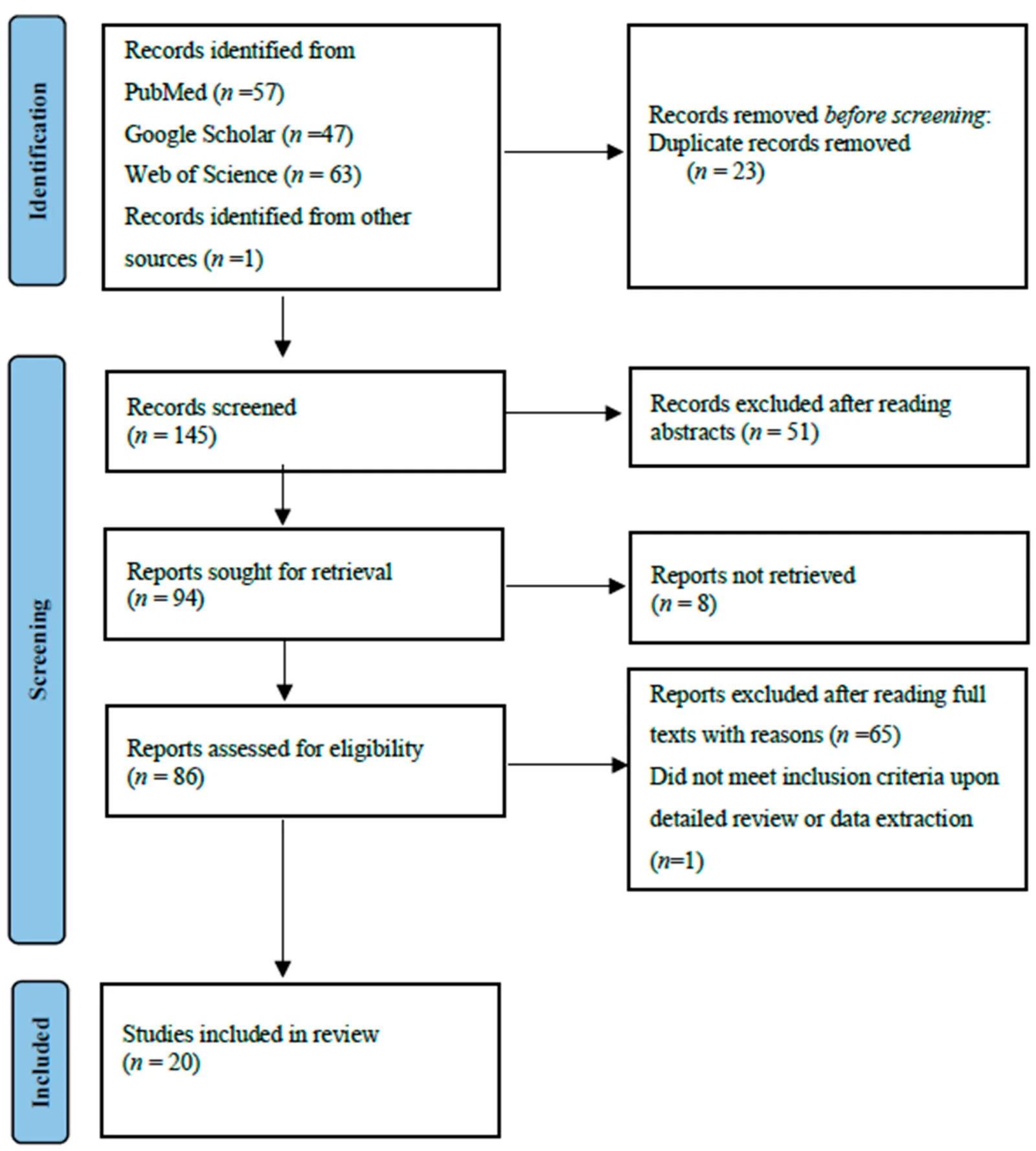
PRISMA 2020 flow diagram for the literature search.

The protocol for the systematic review was managed through Covidence, and a literature search had been performed with databases including PubMed, Web of Science, and Google Scholar starting on 15 May 2025. The process of conducting the systematic review was in accordance with the Preferred Reporting Items for Systematic Reviews and Meta-Analyses (PRISMA) 2020 guidelines (Figure 1). The PRISMA guidelines were followed strictly, and the checklist was attached as a Supplementary File. The search strategy was consulted with a librarian with the syntax of search terms adapted to each database. The following keywords were used: cognitive impairment, cognitive dysfunction/prevention and control, behavior, addictive/prevention and control, substance use disorder, addictive/rehabilitation, and recovery (see the Supplementary Materials for a full search strategy). An article would be eligible if the study design has the following components: 1. People have a current or a history of SUD (population); 2. treatment or rehabilitation procedure for SUD (intervention); 3. a control group with people without SUD or abstinence (comparison); 4. validated cognitive assessments (outcome).

In total, 168 abstracts were identified from the search query from all databases and imported into Covidence. Twenty-three abstracts were removed due to duplication, and 145 remained for being screened. Studies were excluded if they met one or more of the following criteria: non-English language publications; inaccessible full-text articles; absence of a cognitive assessment; study without a documented substance use disorder; if cognitive impairment was attributable to a primary cognitive disorder unrelated to substance use; non-human subjects. Two reviewers were assigned for every abstract, with a third reviewer resolving disagreements in group consultations.

After the first round of screening, 51 abstracts were removed based on whether they were relevant to the study’s scope for population, exposure/intervention and cognitive disorder (i.e., an article included SUD but did not have a cognitive function assessment was excluded). This left 94 studies to be sought for full-text retrieval, but 8 studies did not pass this phase either due to paywall restrictions or inaccessible archives. With the full-text review, a total of 86 reports were assessed. For these 86 articles, 66 were excluded for not meeting inclusion criteria, and the remaining 20 articles were extracted and analyzed (Figure 1). Quality/risk of bias were assessed using the Newcastle–Ottawa Scale [6] or RoB 2 [7]. All observational studies scored between 10 and 14, indicating moderate to high methodological quality or low to moderate risk of bias (Supplementary Table S1). Similarly, all clinical trials have low risk of bias (Supplementary Table S2).

## 3. Results

### 3.1. Prevalence of Cognitive Impairment Found in People with SUD

Cognitive impairment is common among older adults enrolled in treatment for SUD and may interfere with independence and rehabilitation engagement. The prevalence of cognitive impairment has been reported by studies performed in different countries (Table 1). One study recruited 99 participants from Sydney, Australia, with some (*n* = 30) receiving alcohol treatment and others (n = 69) receiving opioid treatment. Cognitive function was assessed with both MMSE and Addenbrooke’s Cognitive Examination-Revised (ACE-R) [8]. In 2015, Lintzeris et al. reported that severe cognitive impairment was identified in 8% of participants when a cutoff score < 24/30 for the MMSE was used compared with a prevalence of 41% when a cutoff score of <82/100 was used for the ACE-R [8]. Using the same dataset, Monds et al. reported in 2017 that the prevalence of MCI was 65% [9]. In 2018, Pelletier et al. [10] reported that 31.1% MCI and 17.8% moderate to severe cognitive impairment were observed in French participants with alcohol use disorder (AUD) [10]. MoCA and Brief Evaluation of Alcohol-Related Neuropsychological Impairments (BEARNI) were used as screening tools of cognitive impairment after a withdrawal of alcohol use for 7–10 days. The average alcohol consumption of the participants was 260 ± 120 g/day. The average starting age of AUD was 33.1 ± 11.8 years, and the duration of heavy drinking was 15.6 ± 10.3 years. Notably, the participants with AUD also used other drugs such as cannabis, cocaine, and/or heroin besides alcohol [10]. Thus, the prevalence of cognitive impairments in the study reflected polysubstance uses instead of alcohol only. In 2019, Allan et al. assessed cognitive function for people with SUD who lived in a residential rehabilitation program in the United States of America (U.S.A.) [11]. For all screened residents (n = 67), the prevalence of cognitive impairment was 49.25% using the ACE-R as the screening tool. In 2020, Richter et al. reported the prevalence rate of cognitive impairment was 54.8% in an Israeli group of polysubstance users [12]. In 2021, Cimarolli et al. found the prevalence rate of cognitive impairment was 23% in 271 participants from an American Geriatric Substance Recovery Program [13]. They also found that SUD adversely affected cognition, and impaired cognition hinders recognition of substance use-related problems or motivation to seek treatments [13].

### 3.2. Cognitive Domain-Specific Effects in People with SUD

Individuals with SUD may use one substance only or multiple substances concurrently. Several studies focused on the effects of single substance use on cognition (Table 2). In one study, all participants recruited from indigenous Australian communities, petrol sniffers (n = 50) had poorer performance on psychomotor, visual attention, learning, spatial awareness, and executive function than their healthy counterparts (n = 96) [14]. Notably, it is common for petrol sniffers from indigenous communities to use more than one substance such as cannabis or alcohol concurrently.

To evaluate how alcohol affects prospective memory, adults with varying levels of consumption (n = 80) were recruited from an alcohol counseling service in the United Kingdom [15]. Participants (n = 60) were divided into no drinking, light (1–9 units/week), medium (10–25 units/week), and heavy (26+ units/week) drinking groups, while the abstinent group had a mean alcohol use duration of 15.7 years (n = 20), prior to the cessation. Chronic heavy alcohol users had impaired prospective memory, potentially affecting daily functioning and treatment adherence even after abstinence [15]. Another study on alcohol recruited two groups of participants: healthy control (n = 20) and alcohol users (n = 20) in Belgium [16]. Sex, age, and education level were matched between the two groups. Participants with AUD had poorer accuracy and a slower reaction time compared to healthy individuals as controls. In addition, inhibitory control was impaired in the participants with AUD.

The effects of tobacco on attentional network functioning were investigated in young adult smokers, focusing on executive control deficits [17]. Participants were recruited from college students from UCLouvain, Belgium, which were divided into groups of healthy controls, light smokers, and heavy smokers (10–25 cigarettes/day). Tobacco use selectively affected executive attention. Heavy smokers demonstrated a specific impairment in the executive attention, while alerting and orienting attentional network preserved.

For methamphetamine users, their cognitive functions were assessed after acute withdrawal as well as over the course of 6 months of abstinence [18]. The participants (n = 106) were recruited from two inpatient rehabilitation centers in Germany. Continued long-term methamphetamine use was associated with poor cognitive performance in areas of attention, concentration, working memory, and cognitive flexibility. By contrast, a recovery of cognitive functions was observed with sustained abstinence. In summary, different cognitive domains were affected by the substance used, and being abstinent can be effective for cognitive function recovery.

How polysubstance use is associated with cognitive changes were also reported in a few studies (Table 2). Cognition was compared between polysubstance users (n = 151) and healthy controls (n = 56) in an Italian study [19]. A range of psychoactive substances were used by the participants (individuals who drink alcohol as the only substance used were excluded). The Battery for Executive Functions in Addiction (BFE-A) has seven short tests measuring memory, attention, flexibility, and self-control. Compared to controls, polysubstance users performed worse on almost all tests, with the most prominent problems in verbal memory and attention. Longer abstinence predicted better delayed recall, while longer histories of drug use predicted slower attention [19]. As expected, a German study reported that significant impairments in attention, psychomotor speed, and executive functioning were observed in polysubstance users [20].

### 3.3. Effects of Rehabilitation on Cognition for People with SUD

The rehabilitation for people with SUD can be in different modalities: abstinence only, targeted medications, and cognitive remediation therapy (CRT), which can be used alone or in combination. Next, how rehabilitation/treatment affects cognitive functions for people with SUD were examined and reported in five studies (Table 3). It was reported that early rehabilitation could enhance engagement and help with the recovery of cognitive impairment for people with SUD [21]. In the study, all participants (n = 72) had an extensive history of SUD (mean = 165.9 months) with an average of 39 days of abstinence before admission. The cognitive rehabilitation group demonstrated faster cognitive recovery and more efficient cognitive functioning than the control groups.

The following two studies focused on the treatment effects of methadone on cognitive functions. For heroin users, methadone maintenance therapy was associated with poorer cognitive performance than those who were abstinent [22]. Male participants (n = 46) were recruited from treatment centers in Granada, Spain. Participants had been stabilized on methadone (n = 23) for at least 15 days with an average dose of 83.8 mg/day or maintained abstinence for at least 15 days (n = 23). The two groups were matched on education, pre-morbid IQ, and employment status, though methadone maintenance group reported a longer history of heroin and cannabis use than the abstinence group, which could be a confounding factor for the findings. With a randomized, nonblinded clinical trial done in Germany, participants with opioid dependence exhibited marked cognitive impairments compared to healthy controls [23]. Participants with opioid dependence were treated with methadone (mean dose = 83 mg/day) (n = 24) or buprenorphine (12 mg/day) for at least 14 days (n = 22). After 8 to 10 weeks, concentration and executive functions were significantly improved.

For the effects of CRT on participants with SUD, one randomized clinical trial was done in the U.S.A. [24]. Participants (n = 48) were randomized into either the cognitive remediation group or the control group. Mean duration of abstinence at baseline = 40.15 ± 65.10 days, with neurocognitive testing deferred until the participant had at least 7 days of sobriety or abstinence. CRT was associated with significant improvements in cognitive function for domains of working memory and executive function. A non-randomized trial was conducted in Australia for studying the effects of CRT on cognition in a residential treatment setting for SUD [25]. Participants were put in the cognitive remediation group (n = 34) and the control group (n = 31) respectively. The cognitive outcomes were improved in both groups at 2 months and maintained at 6 months for outcomes related to executive function. However, no cognitive benefits were observed in the cognitive remediation group compared to the control group.

### 3.4. Improved Cognition Promotes Rehabilitation for People with SUD

It is important to incorporate cognitive assessment in the treatment/rehabilitation process for people with SUD. The impaired cognition in people with SUD, if untreated, would adversely affect their willingness to participate in treatment/rehabilitation (Table 4). On the other hand, if impaired cognition were improved, these with SUD might understand the benefits better and improve the motivation to participate in the treatment/rehabilitation process. It has been shown that better cognitive scores predicted higher abstinence rates, employment, and lower relapse rates with cognitive status mediating 15–25% of treatment/rehabilitation outcomes [12].

There were two American studies on how SUD affected the executive function. The first one was done with 258 heroin and/or cocaine users [26]. Participants reported 9.1 years of regular substance use on average, and about half were polysubstance users. The Wisconsin Card Sorting Test (WCST) was used for assessing problem-solving and executive function. A poor performance on the WCST was associated with a low recognition of substance-related problems [26]. Impaired executive function hindered the motivation of substance users to seek and/or enter treatment. In 2012, another study reported poor executive performance among 60 adults with histories of homelessness and SUD [27]. The poor executive performance may adversely affect their ability or willingness to seek treatment/rehabilitation.

## 4. Discussion

In this report, we analyzed the relationship between single, or polysubstance use and measured cognitive function. Nonetheless, the studied substances, including tobacco, were found to be associated with poor cognitive performance in at least one cognitive domain. Each study found that the cognitive performance was worse in the substance use group than the control group. For example, tobacco smokers were found to have impaired executive control of attention [17] while petrol sniffers had impaired function in spatial awareness and learning [14]. Heavy alcohol users reported having impairments in short-term memory, accuracy, and showed poor reaction time [15,16]. Psychoactive substance users exhibited deficits in verbal memory and attention [19]. Lastly, methamphetamine users demonstrated poor performance in attention, concentration, and reaction times [18].

When more than one substance was used, the cognitive impairment worsened. For instance, when individuals with alcohol use only were compared to individuals who were polysubstance users, the polysubstance user group performed significantly worse in cognitive functions such as auditory–verbal memory and impulsiveness [28]. Another predictor of cognitive impairment for people with SUD was the duration of use. The longer the duration, especially if polysubstances were used, the worse the cognitive performance. This is supported by the finding from a study that the three main predictors for low MoCA scores were age, educational attainment, and length of substance use [29]. It was reported that worse attention, concentration, and reaction time scores were associated with a longer methamphetamine use [18]. Long-term substance use can cause the brain’s microglia and astrocytes to mount an immunological defense. This neuroinflammation can cause damage to the brain and progress into widespread neural dysfunction [30].

Some studies conducted trials by comparing targeted medications or cognitive remediation therapy to abstinence only. Heroin users with methadone maintenance treatment had poorer cognitive performance than the abstinence only group [22]. It is worthy to note that the methadone maintenance group had a longer history of heroin and cannabis use than the abstinence group. However, side effects from the medications could not be excluded for affecting cognitive performance. By contrast, pairing a rehabilitation program with a cognitive remediation therapy showed marked improvements in the speed of cognitive recovery in working memory and executive function [21,24]. This infers that the better the cognitive function, the more likely the person will remain abstinent. However, Allan et al. did not observe any cognitive benefits for the cognitive remediation group over the control group [25]. It is worth noting that it was not a randomized trial, and the sample size was relatively small. High-quality randomized controlled trials are needed to ascertain the effects of treatment/rehabilitation of SUD on cognition.

Much like how the duration of SUD increases the odds of cognitive impairment, the length of abstinence had been associated with an increased odds of cognitive improvement as identified from a few studies. A longer abstinence predicted a better delayed recall [19]. Further, sustained abstinence was found to be associated with cognitive recovery in the case of individuals with methamphetamine dependence [18]. Long-term abstinence itself may be able to drive cognitive improvement by avoiding the harmful effects of used substance(s). However, another possibility exists that these who can maintain long-term abstinence may have a greater inherent capacity for cognitive recovery.

This review report has limitations. First, most of the included studies had an observational, often cross-sectional design. Some studies had relatively small sample sizes. Second, the review covered a wide range of substances, study populations with a variety of clinical and sociodemographic features, and different neuropsychological assessments. Third, confounders such as age, attained level of education, psychiatric comorbidities, traumatic brain injury, depression, and medication use may bring bias to observed associations between SUD and cognition. Last, the literature search was restricted to English-language publications, excluding inaccessible full texts, omitting grey literature, which may bring bias to our overall discussion/conclusion.

In the future, more research needs to be done on the role of cognitive remediation therapy. A person with impaired cognitive function might not fully understand the problem they have but a person with remediation therapy might realize the benefits of staying abstinent. Therefore, rehabilitation programs for people with SUD should focus on improving the impaired cognitive function to have more success in treatment participation and long-term recovery outcomes.

## Figures and Tables

**Table 1:** Prevalence of cognitive impairments in people with SUD.

References	Country	Study Design	Sample Characteristics	Substance Type	Cognitive Function(s)	Cognitive Test(s)
[8]	Australia	Cross-Sectional	99 participants (77% male), age: 55 ± 4.5; education: 11.9 ± 3.2 years	Alcohol (44%); opioids (13% heroin); benzodiazepines (41%); Cannabis (38%)	40% scored <83 on ACE-R (severe impairment); 8% scored <24 on MMSE.	ACE-R; (Addenbrooke’s Cognitive Examination -Revised) MMSE
[9]	Australia	Cross-Sectional	99 participants Age: 55 ± 4.5 years; 77% male; education: 11.9 ± 3.2 years.	69 opioid therapy, 30 alcohol treatment	MCI (65%) and severe impairment (41%); most pronounced deficits in attention/orientation and memory	ACE-R
[10]	France	Cross-Sectional	90 participants (age: 48.9 ± 9.6; 74% male; 79% Had no more than 12 years of education	Alcohol	31.1% mild cognitive impairment, 17.8% moderate to severe cognitive impairment	MoCA (Montreal Cognitive Assessment); BEARNI (Brief Evaluation of Alcohol-Related Neuropsychological Impairments)
[11]	United States of America	Phenomenography	12 participants Age: 34.5 ± 14.3 years Male: 75% Education: 16 ± 2.2	alcohol (42%), cannabis (25%), amphetamines (25%), and opioids (8%)	For all screened residents (n = 67), 49.25% have cognitive impairment Most affected domains: attention/orientation, memory, and executive function (fluency).	ACE-R
[12]	Israel	Cohort	43 participants (age: 40 ± 9; 69.8% male; diverse ethnicity)	heroin/opioids (40%), alcohol (25%), cocaine/stimulants (20%), and cannabis (15%)	54.8% had cognitive impairment	Clock Drawing Test (CDT); MMSE
[13]	United States of America	Cross-Sectional	271 participants (Age: 68.1 ± 8.3; 65% male; 51% White, 29% Black, 19% Hispanic)	Most participants (91%) reported alcohol misuse, 10% prescription misuse, and 17% illicit drug misuse	23% of participants have cognitive impairment	Brief Interview for Mental Status (BIMS)

**Table 2:** Effects of substance use (single or polysubstance) on cognition.

References	Country	Study Design	Sample Characteristics	Substance Type	Cognitive Function(s)	Cognitive Test(s)
[14]	Australia	Cross-Sectional	96 healthy controls and 50 petrol sniffers Age: 16.26 ± 4.07 (range 11–25), 43% male for the control group; sniffers’ age: 17.35 ± 3.65 (range 11–25), 76% male for the sniffers group Education: 7.44 ± 2.55 years (range 4–13) for the control group; 7.92 ± 2.41 years (range 0–12) for the sniffers	Petrol sniffing (regular or episodic use within past 60 days)	Petrol sniffers had poorer performance on psychomotor, visual attention, learning, spatial awareness, and executive function their indigenous counterparts.	CogState computerized battery
[15]	United Kingdom	Cross-Sectional	80 adults (modal age 31–35; ≈50% male);	Alcohol	Heavy drinkers and abstinent participants showed more short-term memory lapses than those who use less alcohol or none.	Prospective Memory Questionnaire
[16]	Belgium	Cross-Sectional	20 participants with AUD and 20 healthy subjects matched with sex, age, and education level.	Alcohol	Poorer accuracy, slower reaction time were seen in participants with AUD than the healthy controls. Impaired inhibitory control was seen in the participants with AUD as well.	Computerized real-life activity test
[17]	Belgium	Cross-Sectional	75 participants (age: 21.8 ± 2; 34.7% male)	Cigarettes	Executive attention was adversely affected by tobacco use.	Attention Network Test
[18]	Germany	Longitudinal (Baseline + 6-month Follow-up)	108 participants 79.6% male; age: 31.67 ± 7.7 (18–56) Education: 73 (67.6%) left secondary school after 6 years or less; 28 (25.9%) completed at least 10 years; 3 (2.8%) had a high school education	44 were primary methamphetamine users and 62 were diagnosed as polysubstance users including methamphetamine	Reduced attention, concentration, and slower reaction times were associated with longer methamphetamine use. After six months of abstinence, significant improvements were observed in attention and processing speed and cognitive flexibility.	Cognitrone, Trail Making Test (TMT), Stroop, N-back, Raven’s Matrices
[19]	Italy	Cross-Sectional	Substance use group (N = 151; Age = 41.7 ± 10.0; 77% male; education: 12.3) Healthy Control Group (N = 56; Age = 36.0 ± 13.8; 30% male; education: 16.4)	psychoactive substances (People who only drank alcohol were not included)	The substance use group had worse verbal memory and attention than the controls.	The Battery for Executive Functions in Addiction (BFE-A)
[20]	Germany	Cross-Sectional	30 male participants with substance use disorders (mean age = 34 ± 8.6 years; 70% completed 9 years of secondary school	Polysubstance use (80%) including stimulants, cannabinoids, alcohol, and opioids	Participants had slower reaction and movement times and reduced cognitive flexibility.	Cambridge Neuropsychological Test Automated Battery

**Table 3:** Effects of substance use treatment on cognition.

References	Country	Study Design	Sample Characteristics	Substance Type	Cognitive Function(s)	Cognitive Test(s)
[21]	United States of America	Randomized controlled trial	72 participants Age: 29.3 ± 6.0, education: 11.9 ± 1.3; 74% male. Race: 56% White, 31% Black, 12% Hispanic, 1% American Indian.	Cocaine (40%); alcohol (25%); opioids (17%); stimulants (4%); cannabis (4%); polysubstance (10%)	Cognitive rehab group improved faster than controls.	Wechsler Adult Intelligence Scale (WAIS) Vocabulary; WAIS Digit Symbol; WAIS Block Design; Category Test; Tactual Performance Test; Trail Making Test Part B
[22]	Spain	Case Control Study	46 men Age = 32.3 ± 5.8; Education: 8.78 ± 2.07 for the AHA group; 9.24 ± 3.91 for the MMP group.	Heroin	Methadone maintenance patients showed worse performance on processing speed, visuo-spatial attention, cognitive flexibility, and working memory and reasoning than patients on the heroin abstinent group.	FAS, animals, fruits; WAIS Letter–Number Sequencing; Oral Trails; WAIS Similarities; Stroop; Five Digit Test)
[23]	Germany	Randomized clinical trial	59 patients (30 on Methadone, 29 on Buprenorphine; 34.5 ± 7.4 years old; 78% male) and 24 matched healthy controls (78% male) Education: 10.5 ± 2.1 years	Opioid dependence (methadone or buprenorphine); 61% also tested positive for other substances such as cannabis, benzodiazepines, or additional opioids	Compared to controls, both patient groups had worse cognitive performance in multiple domains. Modest improvements in concentration and executive functions were observed after 8–10 weeks.	d2-Test of Attention; Regensburger Word Fluency Test (RWT); Verbal Learning and Memory Test (VLMT); Trail-Making Test
[24]	United States of America	Randomized clinical trial	48 veterans (age: 52.6 ± 8.6; 94% male; 24 African American; 20 White; 3 American Indian/Alaska Native; 1 multiracial)	Alcohol use disorder (29 patients); cocaine use disorder (10); opioid use disorder (6); polysubstance use disorder (5)	Cognitive remediation therapy combined with structured vocation therapy works better for recovering cognitive function in some domains (working memory and executive function) than the work therapy alone.	Wechsler Test of Adult Reading. Identical Pairs, Trail Making Test Part A. WAIS-III Digit Symbol Coding, WAIS-III Symbol Search. WAIS-III Digit Span, WMS-III Spatial Span. Hopkins Verbal Learning Test–Revised. Brief Visuospatial Memory Test–Revised. Wisconsin Card Sorting Test, Neuropsychological Assessment Battery Mazes
[25]	Australia	Non-randomized trial	65 participants (age: 37.9 ± 10.7; 59% male; education: 10.0 ± 2.4; 28% Indigenous Australian	Alcohol (46%), amphetamines (40%), cannabis (9%), and other “the symptoms and effects depend on the amount and type of used substances (5%)	About 51% of participants were cognitively impaired at baseline, especially in processing speed. Both CR + TAU and TAU improved cognition at 2 months with executive function gains maintained at 6 months	Brief Assessment of Cognition (BAC), Wisconsin Card Sorting Test (WCST), and Barkley Deficits in Executive Functioning Scale (BDEFS)

**Table 4:** Impaired cognitive function hinders treatment/rehabilitation.

References	Country	Study Design	Sample Characteristics	Substance Type	Cognitive Function(s)	Cognitive Test(s)
[26]	United States of America	Cross-Sectional Study	258 heroin and cocaine users (mean age: 39.1 ± 9.2; 70% male; 59% White; nearly half had less than a high school education (11.1 ± 2.2)	Heroin and/or cocaine	Poor WCST performance	WCST
[27]	United States of America	Cross-Sectional Study	60 participants with a substance use history (mean age: 45.2 ± 10.8; 73% male; race: 49% African American, 29% Hispanic, and 20% White)	Not specified	Poor executive function	Executive Function Performance Test (EFPT)

## Data Availability

All related data are shared in the article. The literature search strategy is part of the Supplementary Files.

## Supplementary Materials

The following supporting information can be downloaded at: https://www.mdpi.com/article/10.3390/life16091443/s1, Table S1. Newcastle-Ottawa Scale (NOS) Quality Assessment of Included Observational Studies; Table S2. RoB 2 Quality Assessment of Included Clinical Trials.

## Abbreviations

The following abbreviations are used in this manuscript:

SUD	Substance use disorder
MMSE	Mini-Mental State Examination
MoCA	Montreal Cognitive Assessment
MCI	Mild Cognitive Impairment
PRISMA	Preferred Reporting Items for Systematic Reviews and Meta-Analyses
ACE-R	Addenbrooke’s Cognitive Examination-Revised
AUD	Alcohol Use Disorder
BEARNI	Brief Evaluation of Alcohol-Related Neuropsychological Impairments
U.S.A.	United States of America
CDT	Clock Drawing Test
BIMS	Brief Interview for Mental Status
TMT	Trail Making Test
BFE-A	The Battery for Executive Functions in Addiction
WAIS	Wechsler Adult Intelligence Scale
RWT	Regensburger Word Fluency Test
VLMT	Verbal Learning and Memory Test
BAC	Brief Assessment of Cognition
WCST	Wisconsin Card Sorting Test
BDEFS	Barkley Deficits in Executive Functioning Scale
EFPT	Executive Function Performance Test
