# Supplementary material for: Substance Use Disorder and Cognitive Impairment
Source: Life (Basel). 2026 Aug 30;16(9):1443. doi: 10.3390/life16091443 (PMC13608821; doi:10.3390/life16091443)
Supplement: Supplementary file 1 [file life-16-01443-s001.zip › life-4500668-supplementary.pdf]

**Studies**		**Sample Selection**				**Comparability**		**Outcome**		**NOS Total Score (Quality Assessment) (0-16)**
	Clearness of Sated Aim (0-2)	Sample Representativeness (0-2)	Sample Size (0-2)	Non-respondents (0-2)	Exposure Assessment (0-2)	Control of Confounding Factors (0-1)	Comparability of Participants (0-1)	Assessment of Outcome (0-2)	Statistical Tests (0-2)	
[6]	2	2	2	0	2	1	0	2	2	13
[7]	2	2	2	0	2	1	0	2	2	13
[8]	2	2	2	0	2	1	0	2	2	13
[9]	2	1	1	0	2	1	0	2	2	11
[10]	2	2	2	1	2	1	0	2	2	14
[11]	2	2	2	0	2	1	1	2	1	13
[12]	2	2	2	0	2	1	0	2	2	13
[13]	2	2	2	0	2	1	1	2	2	14
[14]	2	1	1	0	2	1	1	2	2	12
[15]	2	2	2	0	2	1	0	2	2	13
[16]	2	2	2	0	2	1	0	2	2	13
[17]	2	2	2	0	2	1	0	2	2	13
[18]	2	1	1	0	2	0	0	2	2	10
[20]	2	1	1	0	2	1	0	2	2	11
[24]	2	2	2	0	2	0	0	2	2	12
[25]	2	2	2	0	2	0	0	2	2	12

Table S1. Newcastle-Ottawa Scale (NOS) Quality Assessment of Included Observational Studies. Total score: 13–16 points (high quality and low risk of bias), 9–12 points (moderate quality and moderate risk of bias); <9 points (low quality and high risk of bias).

	Risk of Bias Domains					
**Studies**	**D1**	**D2**	**D3**	**D4**	**D4**	**Overall**
[19]	+	+	+	+	+	+
[21]	-	+	+	+	+	+
[22]	-	+	+	+	+	+
[23]	X	+	+	+	+	+

- Some concerns + Low X High

D1: Bias arising from the randomization process.

D2: Dias due to deviation from intended intervention.

D3: Bias due to missing outcome data.

D4: Bias in measurement of the outcome.

D5: Bias in selection of the reported result.

Table S2. RoB 2 Quality Assessment of Included Clinical Trials.
